# Spotlight on Cancer Informatics

**Published:** 2007-02-04

**Authors:** Constantin Alifieris

**Affiliations:** Assistant Professor, Department of Biomedical Informatics, Vanderbilt Medical Centre, Vanderbilt University. Email: constantin.aliferis@vanderbilt.edu

*Spotlight on Cancer Informatics is a new feature of Cancer Informatics designed to help build a sense of community among informatics researchers attempting to at once provide informatics services to cancer studies while simultaneously conducting independent research that leads to advances in informatics solution. Individuals are “Spotlighted” at the invitation of the Editor-in-Chief. To be considered for an invitation, please send a brief description of your research interests to caninfo@la-press.com along with your curriculum vitae*.

**Figure:**
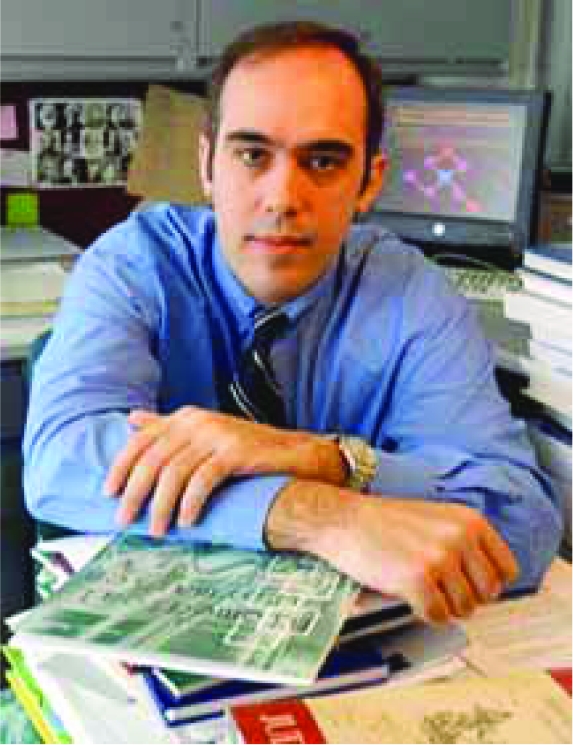
Constantin Alifieris

**Q:** What would you say is the primary focus of your research effort (how do you refer to your ‘sub-area’)?

**A:** The primary focus of my group’s research is developing, validating, applying and automating algorithms for decision support and discovery for molecular medicine. The emphasis is on cancer; however, the methods are broad enough to be useful for a number of other diseases, as well as for general pattern recognition and discovery tasks.

One set of these algorithms is designed to produce models for diagnosis, clinical outcome prediction and personalized treatment decisions. Another set is designed to select compact sets of biomarkers. Finally the last set seeks to discover structural relationships (e.g., gene regulatory networks and protein-protein or gene-protein interaction networks) to shed light on complex molecular mechanisms of disease.

**Q:** What do you consider to be the most significant open questions and research challenges in cancer informatics?

**A:** There exist numerous challenges of which I mention just a few: Overfitting and other data analysis problems caused by very small samples and large dimensionalities. Developing mechanistic (causative) models and separating them from purely predictive ones. Utilizing a multiplicity of information/data types concurrently, for example mass throughput, clinical, imaging, literature, data etc. There is a need for - but lack of standards in - data analysis for mass throughput data. Biologists, physicians and informatics researchers often do not share the same language, scientific culture, and expectations. There are serious assay reproducibility issues. It is not clear how to optimally deliver the results of molecular medicine to physicians at the bedside. Regulating molecular medicine modalities for safety without impeding timely progress in the field is a big challenge. Exploring proper ways to build and maintain interdisciplinary teams and assign academic credit is another. Finally there is a myriad of thorny ethical issues surrounding the storage, protection, and retrieval of patient data.

**Q:** What do you consider to be the most significant developments arising from research in cancer informatics?

**A:** The emerging ability to perform early diagnosis, personalize treatments, predict clinical outcomes, and understand disease using mass-throughput information.

**Q:** Tell us about your collaborative research. How much of your effort is typically focused on helping to provide cancer researchers with clinically significant results?

**A:** Approximately 50% of my work is application of methods and analysis of data for cutting edge biomedical (mainly cancer) research. The rest is methods development, service, and teaching, which I hope to also benefit cancer researchers down the road.

**Q:** Do you find balancing all of these activities challenging? How might cancer centers better meet the increasing demand for the analysis of high-dimensional data?

**A:** It is very challenging as often methods are not well-developed and method development has to take place during the lifecycle of the specific cancer research project, subject to many practical constraints (time, resources, etc.). Furthermore, when a good method is developed, in the initial phases of its lifecycle its application is often not straightforward unless the inventor is involved, which in turn precludes scaling-up of the method development efforts and dissemination of the methods rapidly.

There is a number of ways to meet these needs:

– Developing a critical mass of dedicated experts inside the institution and solid working collaborations with experts in other institutions.
– Creating dedicated groups to develop, test, and automate new methods (an example methods group is the Discovery Systems Laboratory at Vanderbilt) as well as groups that apply appropriate methods and protocols for analysis in research (such a group is the High-Dimensionality Core at the Vanderbilt Ingram Cancer Center).
– Creating consulting services (e.g., the “Omics” clinic organized jointly by the Dpts of Biostatistics and Biomedical Informatics at Vanderbilt) that will disseminate methodological knowledge and connect cancer biology researchers with methods researchers according to the project needs.
– Creating and sharing robust analysis protocols and automated data analysis systems, and code libraries (e.g., the GEMS system for gene expression analysis and the Causal Explorer system for biomarker and regulatory network discovery).

**Q:** What do you consider to be the most pressing challenges or barriers to success in the field of cancer research?

**A:** Cancer is a hugely heterogeneous class of diseases that affect cells and organisms in numerous concurrent ways both at the molecular level and at the system level. Thus developing methods to help us understand the pathophysiology of cancer is particularly challenging.

**Q:** What do you consider to be the most significant developments or advancements arising from cancer research?

**A:** Same as previously stated: the emerging ability to perform early diagnosis, personalize treatments, predict clinical outcomes, and understand disease using mass-throughput information.

**Q:** When did you decide to be—or realize that you were—involved primarily in informatics as a research focus?

**A:** In 1985 when I started intensive informatics research while in medical school, and then in 1991 when I entered a PhD program with this specific focus.

**Q:** Do you currently conduct research on diseases other than cancer?

**A:** My work is inherently methodological and as such applies to many diseases. For example, we have been building models with colleagues to predict lab results across the board for all Vanderbilt inpatient population. In the past I have worked on predicting mortality in pneumonia patients, graft failure in post-transplant patients, mortality in patients with syncope, to classify biomedical literature in internal medicine, etc.

**Q:** Tell us about three or four ‘must-have’ essential informatics computing or research resources that you use on a regular basis developed by someone other than yourself or collaborators. Why are these resources so useful, and why do you consider them essential?

**A:** Matlab, because it facilitates very rapid prototyping of code and allows one to focus on the algorithm instead of the language.

The internet through which access to numerous remote sources of knowledge, tools, and colleagues is facilitated.

A collection of library sources very conveniently accessed in the Eskind Biomedical Library building where Vanderbilt’s Department of Biomedical Informatics is housed.

The last two resources are essential because methods work needs to be grounded very solidly on the vast literature (past and present) on computational, mathematical, statistical, and biochemical/medical/biological research and tools. The first resource is essential because it allows a quick transition from the paper/whiteboard to actual experiments and data modeling.

**Q:** What do you think about the development of open access publishing and open access development? How has either changed your perspective on research and development practices?

**A:** I can see both advantages and disadvantages in these paradigms relative to older and more established ones. Open access publishing gives easier and faster access to new knowledge to some members of the community. On the other hand the publishing costs may be prohibitive to some author groups (especially so outside the US and the more industrialized nations). Open access development is leveraged by low-cost and for gratis coding, however for areas where technology is not mature this may lead to dangerous errors in the software for which there is no accountability. This is especially troublesome for medical and for security-sensitive applications (e.g., biosurveillance). Time will tell whether these models are successful. Neither has affected my own work directly so far.

**Q:** What books do you think should be required reading for researchers involved in informatics? In cancer research?

**A:** The list is very wide, I will mention a few books that I open or cite frequently. In the field of informatics: Cormen et al’s “Introduction to algorithms”, Agresti’s “Categorical data analysis”, and Spirtes et al’s “Causation, Prediction and Search” are books that I visit and cite again and again. Mitchell’s “machine learning” gives a clear (but dated nowadays) introduction to the field, Duda et al’s “Pattern Classification” is an excellent textbook and reference, while Herbrich’s “Learning Kernel Classifiers” is probably the single most useful SVM textbook and reference in my bookshelf.

With respect to the field of cancer research, please note that I am conducting methodological research and not biological so my perspective here is certainly skewed compared to the cancer biologist’s. With this caveat stated, I have found “Molecular Biology of the Cell” (Alberts et al), Genes VII (Lewin, currently in the VIIIth edition), and Liebler’s “Introduction to Proteomics” very valuable. Very readable—and brief—first introductions to cancer molecular medicine for interdisciplinary scientists are Ross’ “Introduction to Oncogenes and molecular cancer medicine” as well as Ross’ “Introduction to molecular medicine”. Redei’s “Encyclopedic Dictionary of Genetics, Genomics and Proteomics” is a worthy encyclopedic reference.

**Q:** What books are on your current reading list?

**A:** Many, but I am currently focusing on large parts of Tietz’s Clinical Chemistry and Molecular Diagnostics. (http://www.harcourt-international.com/catalogue/title.cfm?ISBN=0721601898)

**Q:** Do you teach any courses? If so which ones?

**A:** I teach Biomedical Artificial Intelligence and Machine Learning at the graduate level as well as an advanced lab component associated with that course. I also have given directed studies in AI/Machine Learning and Information retrieval and several seminars and tutorials. I am also on the teaching faculty of the Cancer Biology and Clinical Proteomics graduate-level courses at Vanderbilt.

**Q:** List the historical research figures that you think have most influenced how you think about research? Why are these influences significant?

**A:** Many great scientists have influenced my reasoning and have qualities that I admire. I will mention a few. Collectively the ancient Greek scientists were amazing in their ability to exceed the standard of science at the time: for example they computed the circumference of the earth, computed distances between the earth and the sun, laid the foundations of geometry and logic etc. etc.

Jumping ahead a couple of millenia, Niels Bohr was very influential to me because he did not emphasize whether the data were consistent with his biases: as long as the theory (e.g., Copenhagen interpretation of quantum mechanics) was validated experimentally, he accepted it despite it being vastly counterintuitive. Einstein despite all his brilliance was not able to leap mentally that far and insisted (wrongly) on determinism.

David Hume was incredibly powerful intellectually and personality-wise in recognizing and describing the limits of both faith-based doctrines and inductive science. He practically destroyed them both from a philosophical perspective and lived to be happy regardless. Reichenbach is inspiring because he provided, in my assessment, a simple but very convincing account of why we can still pursue inductive generalization successfully despite Hume being correct.

Karl Marx and Adam Smith laid out two still dominant economic frameworks and identified major principles of human economic behaviour. Aristotle and DaVinci were inspiring on account of their breadth and depth of knowledge. Chomsky is inspiring in his dual ability as linguist and contrarian political scientist with deep humanitarian concerns.

Darwin’s colossal mental leap, as well as his meticulous method toward establishing evolution, are astounding. Richard Feynman was inspiring in his balance of scientific ability as well as ability to be a well-adjusted man. He also was one of the clearest thinkers ever to live: his “Lectures on physics” are the clearest science book I have come across so far. Bertrand Russell was inspiring because of his momentous achievement in the meta-theory of mathematics but also because at the same time he was committed to humanitarian values and chose to be imprisoned rather than betray them. On the side he won a Nobel prize for literature. Similarly Herb Simon won a Nobel prize in economics but did most of his work on computer science/artificial intelligence, and political science.

With respect to my own research area, Gregory F. Cooper my former advisor and mentor introduced me to rigorous, principled and uncompromised research. He also taught me Bayesian networks and computational causal discovery, among other topics. Judea Perl, Peter Spirtes, Clark Glymour, Granger, and Cooper are among the major pioneers of modern computational causal discovery. These researchers have collectively shaped a very powerful paradigm for discovery using computational tools and this collective achievement will prove to be no less significant in my opinion than many of the highest achievements of science so far.

**Q:** Could you describe for us briefly what key insights you think researchers in the area of causal discovery have provided that make modern computational causal discovery so exciting to you?

**A:** The main insights are:

– Causation is a crucial aspect of discovery and has to be addressed explicitly when thinking about research and data collection/analysis.
– There is a formal framework that ties together causation and prediction.
– Randomized experiments are not always feasible, ethical, efficient or even correct for discovering causality.
– It is mathematically and algorithmically possible to learn causal relationships from observational data or mixtures of observational and experimental data.
– It is mathematically and algorithmically possible to discover structural confounding (i.e., induce correct causal structure in the presence of hidden (i.e., unmeasured) variables)).
– Although causal discovery is worst-case intractable, there exist algorithmic techniques and reasonable assumptions to make it tractable in practical settings.

**Q:** Which research meetings do you attend on a regular basis? Please provide URL’s any other information you consider relevant.

**A:** I almost never miss the AMIA Fall meeting (www.amia.org). Occasionally I go—or at least send papers—to AAAI (www.aaai.org/Conferences/National/2006/aaai06.html), ISMB (ismb2006.cbi.cnptia.embrapa.br/), ICML (www.icml2006.org/icml2006/14770.html), KDD (www.kdd2006.com/), AI and Stats (www.gatsby.ucl.ac.uk/aistats/), FLAIRS (www.indiana.edu/~flairs06/), MEDINFO (www.medinfo2007.org/), and UAI (www.ics.uci.edu/~csp/uai2006/).

**Q:** Please tell us about your own resource development efforts. Which of your computing resources or research papers would you like most people to know about?

**A:** Currently, the Markov Blanket & Bayesian Network discovery algorithms such as HITON and MMHC for biomarker discovery and structural discovery. Related papers and code can be found in the DSL web site: www.dsl-lab.org. Another is the GEMS system for gene expression modelling and biomarker discovery; papers and code are available from the DSL web site. Another one is the Causal Explorer toolkit for causal discovery and biomarker discovery (code also available from www.dsl-lab.org).

**Q:** If you could change three things about how informatics research is conducted, used, perceived, or resourced, what would they be?

**A:** First, Increase the realization that informatics is less about computers and more about methods. Second, rigorous training in biomedicine *and* computer science, math, and statistics is essential for the advancement of the field. Third, informatics is not just an enabling technology but a field that contributes important novel methods for discovery.

**Q:** What do you think are the most significant cancer research studies in the last year that have been made possible by advances in informatics? Do you have any that specifically stick out in your mind as breakthroughs?

**A:** It is very difficult to make a “most significant” determination for breakthrough clinical and research studies before seeing longer-term clinical impact and citation impact respectively. Moreover, in a sense, all papers were made possible by advances in informatics: regardless of study, it is safe to assume that samples were assayed by computer-controlled machinery, resulting data was analyzed with statistical and pattern recognition software, and results were indexed/stored/retrieved, and published electronically. It is also more than probable that hypotheses were shaped by consulting databases containing bibliographic, sequence, functional, evolutionary, genetic and other stored knowledge.

In terms of excellent—but certainly not “most significant”—specific examples of papers using informatics for cancer research, I could mention include “Gene Expression Tests Foretell Breast Cancer's Future,” Ken Garber, Science 19 March 2004: Vol. 303. no. 5665, pp. 1754 – 1755. This report and the prior research leading to it, is an exciting example of clinical bioinformatics-enabled service that is offered to the public and that has the potential to revolutionize care for cancer patients (by predicting metastases in breast cancer patients and helping treatment decisions).

As a highly-regarded example of array technology enabling discovery of new and significant biological insights about cancer I can mention Westbrook TF, Martin ES, Schlabach MR, Leng Y, Liang AC, Feng B, Zhao JJ, Roberts TM, Mandel G, Hannon GJ, Depinho RA, Chin L, Elledge SJ. “A Genetic Screen For Candidate Tumor Suppressors Identifies REST.” Cell. 2005 Jun 17;121(6):837–48.

